# Ovarian cancer causing hyperprolactinemia: A case report and narrative review

**DOI:** 10.1097/MD.0000000000040585

**Published:** 2025-02-07

**Authors:** Sandra Šakinienė, Džilda Veličkienė

**Affiliations:** a Department of Endocrinology, Hospital of Lithuanian University of Health Sciences Kaunas Clinics, Kaunas, Lithuania; b Faculty of Medicine, Medical Academy, Lithuanian University of Health Sciences, Kaunas, Lithuania; c Institute of Endocrinology, Medical Academy, Lithuanian University of Health Sciences, Kaunas, Lithuania.

**Keywords:** case report, hyperprolactinemia, ovarian cancer

## Abstract

The most common cause of hyperprolactinemia is prolactinoma. In addition, it is necessary to exclude potential physiological and pharmacological factors as well as health disorders to determine the cause of hyperprolactinemia. However, few studies have linked elevated prolactin (PRL) levels to ovarian cancer (OC). OC cells can ectopically release PRL, which then attaches to PRL receptors (PRLRs) in ovarian tissue and initiates signaling cascades that induce OC carcinogenesis. Therefore, we can consider PRL as a biomarker or tumorigenesis factor for OC. Furthermore, both PRL and PRLRs are potential therapeutic targets. A 50-year-old female presented with complaints of breast enlargement, soreness, and hyperprolactinemia, in addition to advanced OC. Hyperprolactinemia along with advanced high-grade serous ovarian carcinoma. Due to the patient’s fear of confined spaces, magnetic resonance imaging of the pituitary gland under general anesthesia was prescribed to rule out pituitary pathology. Magnetic resonance imaging was not performed due to the deterioration of the underlying condition, and the patient died 2.5 years after the diagnosis of OC. Hyperprolactinemia caused by OC is a rare condition for which there is a lack of literature and case studies. PRL produced by OC tissue binds to PRLRs in an autocrine or paracrine manner, initiating signaling cascades that induce OC tumorigenesis. In combination with other biomarkers, PRL may serve as a biomarker for OC. To establish the relation between OC and elevated PRL levels, additional large-scale population studies are required, with diagnostic and treatment procedures coming first.

## 1. Introduction

Prolactinomas are the most common cause of hyperprolactinemia. Medications that increase gastrointestinal motility, antidepressants, antipsychotics, and antihypertensive agents are also most frequently associated with hyperprolactinemia. Pregnancy, breastfeeding, physical stress (such as experiencing pain), and health conditions (such as renal or adrenal insufficiency and hypothyroidism) may also play a role in its development. Nevertheless, in some cases, the cause of hyperprolactinemia remains unknown.^[1]^

Ovarian cancer (OC), also known as the “silent killer,” is the most fatal gynecologic cancer, the 5th leading cause of cancer-related death, and the 11th most common type of cancer in women. Cure and survival rates have not changed significantly despite increased knowledge of OC, as early detection continues to be difficult. This is due to a lack of effective screening tools as well as vague or similar symptoms of other noncancerous conditions.^[2]^

Over the past few years, several reports have suggested that there may be some relations between prolactin (PRL) and OC. PRL is a polypeptide hormone that plays a crucial role in lactation. Numerous other PRL functions have also been identified.^[3]^ The pituitary gland is responsible for the production of a systematic PRL, while other tissues, including the ovaries, produce extrapituitary PRL, which mostly acts in a paracrine or autocrine manner.^[3,4]^ Extrapituitary PRL has a different promoter site than pituitary PRL; however, their amino acid structures and functions are identical.^[5,6]^ prolactin receptors (PRLRs) are also expressed in normal ovarian and fallopian tube tissues.^[3]^ However, OC tissues have a greater number of PRLRs than normal ovarian tissue. In addition, elevated PRL levels were detected in all stages of OC.^[6]^ As a result, elevated levels of PRL and PRLRs in OC tissues indicate the presence of a potential PRL loop that supports tumor growth in an autocrine manner.^[4]^ Studies have shown that PRL increases the survival rate of OC cells by stimulating proliferation and inhibiting apoptosis.^[3,4]^ Since PRL is involved in processes that support carcinogenesis, it has been suggested as a biomarker for the diagnosis of OC.^[4]^

OC-related hyperprolactinemia is a rare disease for which there is a lack of information regarding its pathophysiology and treatment methods. We present a case of a female patient with advanced OC and elevated PRL levels, as well as a comprehensive literature review of probable disease mechanisms, other reported similar cases, and new diagnostic and treatment opportunities.

## 2. Case report

A 50-year-old female patient was consulted by an endocrinologist in the Endocrinology Department of the university hospital for possible hyperprolactinemia. The patient provided written informed consent to allow her health data to be discussed with students and fellow clinicians at her initial session.

The patient’s main complaints were breast enlargement and soreness; other signs included a dry unproductive cough and fatigue. Three years before the patient’s presentation of hyperprolactinemia, she was diagnosed with OC. One year after the diagnosis of OC was confirmed, a left salpingo-ovaroectomy was performed, and the histology of the removed ovarian tissue revealed the presence of high-grade serous ovarian carcinoma pT3bNxMxLVIo. The patient declined immunohistochemical examination of the surgically removed tumor tissue. A computer tomography scan performed after surgery revealed carcinosis and ascites; consequently, radical cytoreduction was unlikely, and chemotherapy with paclitaxel and carboplatin was prescribed. Six cycles of paclitaxel and carboplatin had no effect; therefore, paclitaxel and bevacizumab were administered owing to the recurrence of a platinum-resistant disease. The patient’s condition improved after 2 cycles of paclitaxel and bevacizumab, and she discontinued the treatment. After 4 months, the patient’s condition deteriorated, and the patient desired further treatment; paclitaxel and bevacizumab were continued. In the context of the most recent chemotherapy, the patient complained of breast pain; however, the mammogram and ultrasonography of the breasts ruled out the possibility of breast cancer.

The patient had a family history of oncological disorders since her mother was diagnosed with colon cancer and her aunt (mother’s sister) was diagnosed with OC. A geneticist consulted the patient and confirmed a heterozygous variation in the *BRCA1* gene with uncertain clinical significance.

An objective examination of the patient revealed the following data: height, 164 cm; weight, 56 kg; and body mass index, 20.82 kg/m². By palpation, the grade 1A thyroid gland was soft. The breasts were firm and indurated, with redness covering the middle thirds of both breasts; galactorrhea was not observed. The following sex hormone levels confirmed the presence of menopause: follicular-stimulating hormone, 100 IU/L (postmenopausal women reference range: 25.8–134.8 IU/L); luteinizing hormone, 75 IU/l (postmenopausal women reference range: 7.7–100.6 IU/L); and estradiol, 18 pmol/L (postmenopausal women reference range: <18.4–505 pmol/L). The thyroid ultrasonoscopy detected the nodular thyroid gland (EUTIRADS II), and the thyroid-stimulating hormone concentrations of 0.57 mU/L (reference range: 0.4–3.6 mU/L) resembled normal thyroid function. Two weeks before the endocrinologist appointment, the patient’s PRL levels demonstrated hyperprolactinemia: PRL, 1605 mU/L (reference range: 105–548 mU/L); monomeric PRL, 1150 mU/L (reference range: 74–496 mU/L); and macroprolactin, 151 mU/L (reference range not provided). On the day of the visit, the PRL level rose to 1900.9 mU/L (reference range: 105–548 mU/L), and the macroprolactin level was 43.7% (reference range: 0%–40%). PRL level dropped to 1707.06 mU/L (reference range: 105–548 mU/L) nearly 3 weeks after the initial consultation, spontaneously without treatment.

We confirmed the primary diagnosis of hyperprolactinemia and the associated condition of advanced high-grade serous ovarian carcinoma.

To investigate the etiology of hyperprolactinemia, a review of the chemotherapeutic agents’ characteristics revealed no association with hyperprolactinemia. Due to the patient’s concern about being confined in small spaces, magnetic resonance imaging (MRI) under general anesthesia was planned to rule out pituitary gland pathology. However, the underlying condition worsened, leading to the cancelation of the procedure.

**Figure 1.**
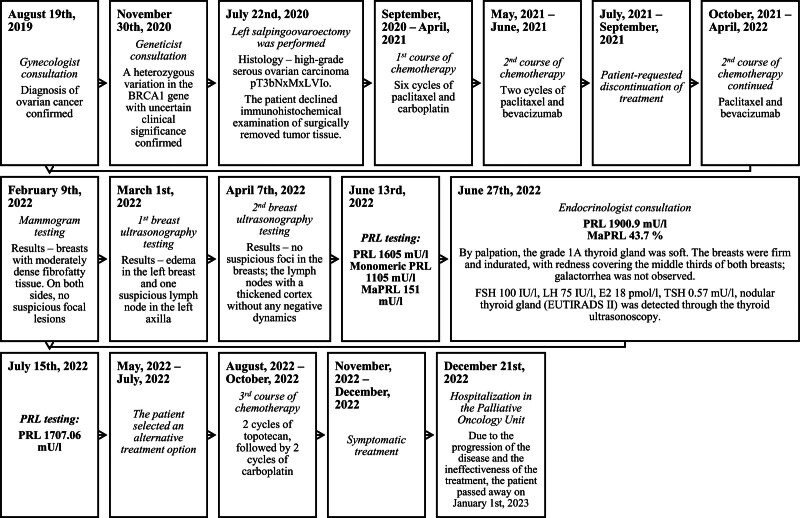
The timetable of events. MaPRL = macroprolactin, PRL = prolactin.

After the final course of chemotherapy with paclitaxel and bevacizumab, the patient received 3 months of alternative treatment. However, the prognosis for the patient was poor: the disease was spreading, the patient’s intolerance complicated the treatment, and no cure was anticipated. Nevertheless, the patient received 2 cycles of chemotherapy with topotecan, followed by 2 cycles of chemotherapy with carboplatin. Regrettably, the combination of topotecan and carboplatin chemotherapy proved to be unsuccessful. The patient experienced severe illness, including high ileus, vomiting for about 20 days, abdominal pain, and oral fungi, leading to the administration of symptomatic treatment. As the patient’s condition continued to deteriorate, she was admitted to the Palliative Oncology Unit. Due to the progression of the disease and the ineffectiveness of the treatment, the patient passed away 2.5 years after the diagnosis of OC was confirmed and 6 months after the diagnosis of hyperprolactinemia (Fig. 1).

## 3. Methods

This narrative review was conducted by collecting published studies and case reports that discussed OC association with ectopic prolactin secretion. A thorough investigation of scientific databases, including PubMed/MEDLINE, Science Direct, and SpringerLink, was conducted to identify relevant articles. The investigation, which extends from the inception of databases to January 2023, utilized a combination of keywords such as “Ovarian Cancer,” “Hyperprolactinemia,” “Ectopic Prolactin,” “Diagnosis,” “Treatment,” “Case Report.” A total of 27 publications and case reports were included and cited in this review.

## 4. Discussion

This clinical case points out the possible significance of PRL in the development and progression of OC. In this research, we attempted to identify if there was ectopic PRL secretion by the OC. Initially, chemotherapeutic drugs were believed to be associated with hyperprolactinemia. However, a review of the drugs’ characteristics revealed that there was no such relation. A brain MRI under general anesthesia due to the patient’s fear of being confined in small spaces was planned to rule out pituitary pathology; however, it was not performed due to the patient’s deteriorated condition. In addition, no autopsy was undertaken after the patient’s death to rule out pituitary gland pathology. Also, there was a lack of data from the patient’s medical history about immunohistochemistry for PRL of removed OC tissue, PRL levels onset of the cancer diagnosis, and its changes over the course of the disease. Lastly, this case did not provide sufficient evidence to confirm ectopic PRL secretion due to OC.

## 5. The literature notes comparable case reports

In the literature, there are few case reports on this condition.

In 1987, the first case report of ectopic PRL secretion resulting from ovarian malignancy was published. It describes a 6-year-old girl with ovarian gonadoblastoma resulting in secondary hyperprolactinemia. PRL-positive immunohistochemistry of removed ovarian tissue, the presence of a PRL concentration gradient between the tumor vein and the peripheral vein, and a decrease in the PRL concentration in the blood after removal of the gonadoblastoma provide evidence of ectopic PRL synthesis in vivo by the ovarian tumor.^[7]^ Case reports of hyperprolactinemia in ovarian malignant small cell tumors of pulmonary type^[8]^ and in juvenile granulosa cell tumors^[9]^ are also presented. In both cases, the pituitary gland appeared normal on MRI, the PRL level dropped after the ovarian tumors were surgically removed, and immunohistochemistry of the removed ovarian tissue showed that the tumors had been positive for PRL. Serum PRL levels in these cases were highly predictive of disease progression.^[8,9]^ In our case, there was no information on the immunohistochemistry for PRL of the removed OC tissue, and there was also no data on PRL levels before and after the surgery or their changes as the disease progressed. In addition, the patient was too ill to undergo an MRI under general anesthesia, making it impossible to rule out the pathology of the hypophysis. It could be assumed that the patient’s hyperprolactinemia was asymptomatic before the spread of the disease and that the presence of metastasis, based on the short PRL half-life, autocrine PRL secretion, and the increased area of tumor distribution, increased the PRL concentration to the point of symptom onset.^[9]^

Ovarian tumors, including ectopic pituitary tissue, have also been linked to secondary hyperprolactinemia; the earliest case report of this was reported in 1990.^[10]^ Ovarian tumors are not the only entities capable of secreting ectopic PRL. Uterine leiomyoma,^[11]^ uterine fibroma,^[12]^ uterine tumors resembling ovarian sex cord tumors,^[13]^ acute myeloid leukemia,^[14]^ metastatic melanoma,^[15]^ and perivascular cell tumors^[16]^ may also produce PRL ectopically.

## 6. Is there a proven relation between PRL and OC? Data from experiments on cell cultures and rodents

According to the findings of the study, individuals diagnosed with OC had increased median PRL levels. Also, PRLRs were expressed in OC tissues up to 98%,^[5,6,17]^ although normal ovarian epithelium was negative for PRLRs^[5]^ or had low levels of PRLRs.^[6,17]^ Thus, PRL binds to its receptor and activates signaling cascades (one of the most important signaling pathways is Janus kinase/signal transducer and activator of transcription (JAK/STAT), thereby activating OC tumorigenesis,^[4–6,18]^ including cell proliferation, angiogenesis, antiapoptosis, chemoresistance, migration, inflammation, and suppression of antitumor immune responses.^[4–6,18,19]^

It was discovered that normal ovarian epithelial cells incubated with PRL demonstrated malignant morphological transformation and that tumors were observed at the injection site in rodents 2 weeks after the subcutaneous injection of these cells. No tumors were found in rodents injected with cells that had not been pretreated with PRL.^[5]^ Karthikeyan et al^[20]^ found that spontaneous epithelial OC model cells incubated in rodents produced subcutaneous tumors, and that transcriptome analysis identified Prl2c2 (a member of the PRL protein family whose structure and function are similar to human PRL) as the most critical protein for these cells’ carcinogenesis in vivo. These data are crucial in determining the PRL’s influence on OC.

## 7. PRL as a biomarker of OC or a factor in tumorigenesis?

Previously published case reports suggest that PRL could serve as a biomarker, as it reflects the progression of the disease.^[8,9]^ Numerous studies in the literature attempt to identify combinations of biomarkers, including PRL, that may be more sensitive for detecting OC. Walker et al^[21]^ examined a combination of 4 biomarkers, including PRL, cancer antigen 125 (Ca125), migration inhibitory factor, and osteoponin, which were more sensitive, specific, and accurate than Ca125 alone in distinguishing OC from healthy controls. The accuracy of the 4 protein biomarkers was 91%, while the accuracy of Ca125 was 85%. The combination of 5 biomarkers, including PRL, Ca125, human epididymis protein 4, osteoponin, and leptin was examined by Hasenburg et al.^[22]^ The latter combination also demonstrated superior differentiation results for OC compared with Ca125 alone.

On the contrary, PRL’s capacity to produce malignant transformation of immortalized normal ovarian epithelial cells and tumor growth stimulation when injecting these cells into rodents provides proof that PRL is a factor in tumorigenesis.^[5]^ Furthermore, PRL and PRLR cooperate to induce tumorigenesis by activating signaling cascades.^[4–6,18]^

## 8. Is there a new diagnostic approach for PRL-positive OC?

A more sensitive, noninvasive molecular imaging method for OC has been developed based on increased PRLR expression in OC and its potential to drive ligand-induced internalization. Sundaram et al^[17]^ utilized human placental lactogen as a ligand to link with PRLR, combining it with the MRI imaging agent gadolinium and the near-infrared fluorescent imaging agent of fluorescence molecular tomography. The results of this investigation disclosed a high sensitivity to detect small tumors (up to 10 mg) and cancer metastases. In addition, malignant tumors were distinguished with high specificity from benign tumors. Before implementing molecular imaging of PRLR-positive cancer cells in clinical practice, additional research is necessary to optimize the human placental lactogen conjugate.

## 9. What are the possible therapy options?

As PRL–PRLR interaction mostly works through the STAT activation cascade, the STAT signaling pathway could serve as a therapeutic target. Wu et al^[19]^ described 2 medications examined in clinical trials. First, tocilizumab, an interleukin 6 receptor inhibitor. Tocilizumab functions by enhancing the patient’s antitumor immunity.^[23]^ Second, ruxolitinib, an inhibitor of JAK1/JAK2, was also well tolerated in clinical trials of phases I and II.^[24]^ Ruxolitinib enhances the antitumor activity of chemotherapeutic agents in epithelial OC.^[19]^

Other studies suggest PRL and PRLR as potential OC treatment targets.^[18,25]^ A PRLR expression blockade could inhibit PRL-caused cancer cell proliferation.^[20]^ Prolactin receptor antagonist (PRLRA), G129R, is a variant of human PRL that differs by a single amino acid and inhibits PRL-induced carcinogenesis signals responsible for the proliferation of malignant cells. G129R functions through 2 distinct mechanisms. First, G129R inhibits the JAK/STAT signaling pathway. In comparison to the control group, the G129R treatment reduced tumor size by 50%. In addition, the combination of G129R and paclitaxel reduced tumor size by 90% compared with the control group. The second mechanism is that G129R induces autophagy in ovarian tumor cells. Antagonism of the PRL–PRLR axis for an extended period led to the formation of late-stage autophagic vacuoles, which resulted in programmed cell death.^[18]^ Another PRLRA, S179D, also effectively reduced cancer cells’ quantity and migration. In addition, inhibiting only PRLRs is insufficient due to negative PRL feedback, which could result in elevated PRL blood concentrations; consequently, dopamine agonists reduce the quantity of circulating PRL levels.^[26]^

There are known to be 3 types of PRLRs: 1 long form and 2 short forms (SF1a and SF1b). Cells expressing more long-form PRLRs exhibited a stronger PRL response. Therefore, we could propose an exact type of PRLR as selection criteria for potentially aggressive tumors, for which PRLRA treatment would be the most effective.^[26]^

A brief half-life of PRLRA is a limitation of PRLRA therapy. However, Yu et al^[27]^ discovered a method for extending the half-life of PRLRA. Researchers created a protein with an albumin-binding domain and PRLRA that binds serum albumin in the blood, thereby lengthening PRLRA’s half-life in vivo.

## 10. Conclusions

Hyperprolactinemia caused by OC is a rare condition, for which there is a lack of literature and case studies. Based on the literature, PRL produced by OC tissue binds to PRLR in an autocrine or paracrine manner, initiating signaling cascades that induce OC tumorigenesis. PRL, in combination with other biomarkers, may serve as a biomarker for OC. To establish the relation between OC and elevated PRL levels in blood serum, additional, large-scale population studies are required, with diagnostic and treatment procedures coming first.

## Author contributions

**Visualization:** Sandra Šakinienė.

**Writing – original draft:** Sandra Šakinienė.

**Conceptualization:** Džilda Veličkienė.

**Project administration:** Džilda Veličkienė.

**Resources:** Džilda Veličkienė.

**Supervision:** Džilda Veličkienė.

**Validation:** Džilda Veličkienė.

**Writing – review & editing:** Džilda Veličkienė.
